# Nonlinear Bayesian Estimation of BOLD Signal under Non-Gaussian Noise

**DOI:** 10.1155/2015/389875

**Published:** 2015-01-26

**Authors:** Ali Fahim Khan, Muhammad Shahzad Younis, Khalid Bashir Bajwa

**Affiliations:** ^1^School of Electrical Engineering and Computer Science, National University of Sciences and Technology, Islamabad 44000, Pakistan; ^2^Department of Electrical Engineering, Institute of Space Technology, Islamabad 44000, Pakistan; ^3^Department of Telecommunication Engineering, University of Engineering and Technology, Taxila 47080, Pakistan

## Abstract

Modeling the blood oxygenation level dependent (BOLD) signal has been a subject of study for over a decade in the neuroimaging community. Inspired from fluid dynamics, the hemodynamic model provides a plausible yet convincing interpretation of the BOLD signal by amalgamating effects of dynamic physiological changes in blood oxygenation, cerebral blood flow and volume. The nonautonomous, nonlinear set of differential equations of the hemodynamic model constitutes the process model while the weighted nonlinear sum of the physiological variables forms the measurement model. Plagued by various noise sources, the time series fMRI measurement data is mostly assumed to be affected by additive Gaussian noise. Though more feasible, the assumption may cause the designed filter to perform poorly if made to work under non-Gaussian environment. In this paper, we present a data assimilation scheme that assumes additive non-Gaussian noise, namely, the *e*-mixture noise, affecting the measurements. The proposed filter MAGSF and the celebrated EKF are put to test by performing joint optimal Bayesian filtering to estimate both the states and parameters governing the hemodynamic model under non-Gaussian environment. Analyses using both the synthetic and real data reveal superior performance of the MAGSF as compared to EKF.

## 1. Introduction

The understanding of neuronal activation of brain, associated with a particular cognitive event, has caused great interest in the neuroimaging community. Functional magnetic resonance imaging (fMRI) modality offers an attractive mechanism to get an insight into the hemodynamic changes occurring inside the brain by exploiting the BOLD signal with high spatial resolution. The de facto method to analyze BOLD signal still remains the statistical parametric (SPM) technique developed by Friston et al. [1]. It attempts to establish a linear relationship between the observed BOLD signal and the underlying physiological processes inside the brain by convolving so-called Hemodynamic Response Functions (HRFs) with a specific design matrix corresponding to the experimental stimulus. Unfortunately, HRFs vary considerably from one part of the brain to the other and from person to person [2, 3]. An approach to battle the variation of HRF across the brain was presented by Gandolla et al. [4] where including the HRF spatial and temporal derivates were able to better capture the HRF inhomogeneities across the brain.

Although neurons in the brain exist at microscopic level, fMRI studies are made at macroscopic level by analyzing composite data generated from a plethora of neurons residing in small regions in the brain called voxels. Attempts have been made to model the BOLD signal by a set of nonlinear, nonautonomous differential equations linking hemodynamic changes with a set of psychological variables. Balloon or simply the hemodynamic model is one such example wherein extending the idea of distending venous chamber [5] and the Windkessel theory [6], Friston et al. establish the neurovascular coupling of physiological variables, namely, neuronal stimulus signal, cerebral blood volume (CBV), cerebral blood flow (CBF), and total deoxyhemoglobin content (dHb) [7]. In an fMRI experiment, each voxel produces a noisy time series data constituting the observation data which is used to invert the hemodynamic model to estimate the states as well as the parameters. Friston et al. calculated the hemodynamic model parameters using a Volterra Kernel series approximation to characterize the hemodynamic response [7]. Later they introduced a Bayesian framework free of Volterra Kernels [8]. Coupled brain regions were included in the model later on [9]. Such type of model inversion is known as dynamic causal modeling (DCM) and is extensively used to study brain connectivity. Though popular, the DCM does not incorporate physiological noise. This assumption is impoverished as studies contend the presence of significant endogenous fluctuations in neuronal activity [10]. Riera et al. went a step further and included physiological noise in their state space stochastic model. More interestingly, they performed blind deconvolution by using radial basis functions (RBS) to estimate the neuronal input signal [11]. Their implementation utilized the local linearization filter (LLF) [12] that closely resembled the celebrated extended Kalman filter [13] for continuous dynamic systems.

To accommodate for the high nonlinearities in the hemodynamic model, Johnston et al., Murray and Storkey and Michah employed the particle filter for model inversion [14–16]. Unlike the Kalman filter family which is a local filter, particle filter is a global filter. It handles the nonlinearities well and offers superior performance but at the cost of high computation. Utilizing the nonlinearity combating capability filter, Hu et al. utilized the square root unscented Kalman (SRUKF) filter to perform joint state and parameter estimation [17]. Later, they proposed a dual UKF filter wherein the states and parameters were estimated separately. The scheme eliminated artificially induced and unwanted covariance between the state and parameter estimates and also offered faster convergence [18]. Havlicek et al. utilized the square root cubature Kalman filter (SRCKF) to perform joint estimation of the physiological states and parameters of the balloon model [19]. A similar technique presented by Hu et al. [20] catered for low frequency drift often found in fMRI scans. Out of the above stated techniques, only Riera and Martin performed blind deconvolution; that is, they estimated not only the states and parameters but also the input signal. Another set of techniques other than Bayesian have been introduced by Friston named dynamic expectation maximization (DEM) and variational filtering [21].

The techniques mentioned above have contributed significantly to the understanding and quantification of the physiological processes responsible for neuronal activation. Unfortunately, these approaches mostly assumed both the process noise driving the dynamic model and the measurement noise affecting the measured data to be Gaussian in nature. Although the assumption offers the attraction of simplicity of implementation at face value, yet it is pernicious. Not surprisingly, real world systems are often plagued by non-Gaussian noises such as impulsive noise, Rayleigh noise, Levy noise, and Cauchy noise and so forth. In some studies, fMRI data has been shown to follow heavy tailed gamma distribution [22] and Rician distribution [23]. Impulsive noise is another common non-Gaussian noise source that is common in fMRI time series [24]. In such an environment, the filters designed for Gaussian noise are susceptible to poor performance and even divergence.

Several strategies may be employed to handle non-Gaussianity. An example is Middleton classes A, B, and C models [25, 26]. Another potent technique presented by Sorenson and Alspach, known as the Gaussian sum filter (GSF), considered both the state noise and the measurement noise to be non-Gaussian [27]. Their assumption was that a series of Gaussian distributions could be combined together to represent the noise sequences. They, however, used a finite truncated series of Gaussian terms to yield an optimal minimum mean square estimator (MMSE) filter. Thus, the GSF had a bank of Kalman filters working in parallel, each filter matched to a single term of the Gaussian sum. A major setback of this approach was the growing computational burden at each iteration step. Thus the method is not computationally feasible in several applications. To mitigate the computational cost, Masreliez and Martin proposed a filter based on the so-called score-function [28, 29]. Although less computationally expensive than the GSF, the proposed strategy required problem dependent nonlinearities and still a computationally expensive procedure to develop the score function.

Adaptive GSF is a filter that is an approximate version of the GSF and can be used without the problem of increasing computational burden [30]. The filter employs a Bayesian learning method to transform a mixture of non-Gaussian terms into a single Gaussian term. Plataniotis and Venetsanopoulos demonstrated the use of AGSF in the application of narrowband interference suppression under non-Gaussian noise [31].

In this paper we attempt to invert the Balloon model in the presence of non-Gaussian measurement noise while still keeping the process noise to be Gaussian. The noise chosen is the *e*-mixture impulsive noise which is a form of Gaussian mixture. A modified adaptive Gaussian sum filter having EKF (MAGSF) working in parallel is employed to jointly estimate the states and parameters of the hemodynamic model. For comparison, the MAGSF is compared with EKF. Subsequent analyses show superior performance of MAGSF as compared to the EKF in non-Gaussian noise.

## 2. Materials and Methods

### 2.1. Hemodynamic Model

**Figure 1:**
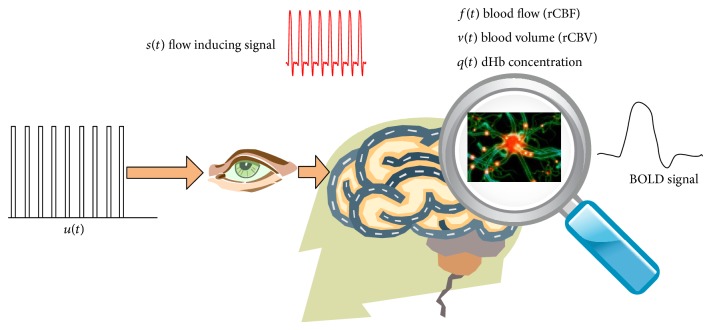
The hemodynamic approach illustration.

The hemodynamic model describes a mechanism that links the synaptic activity to the observed time series fMRI BOLD signal in a voxel. Although several enhancements to the original model have been proposed [32–34], the model initially proposed by Buxton et al., Mandeville et al. and later completed by Friston et al. is sufficient [35].  Figure 1 shows an example of the hemodynamic approach. A human test subject is subjected to a periodic visual stimulus. Consequently, the brain generates a flow inducing electric signal *s*(*t*) that causes blood to flow into a venous compartment fed by blood capillaries inside a region in the brain. This inflatable venous compartment can be further modeled as an input state output system having compartment volume *v*(*t*) and deoxyhemoglobin (dHb) content *q*(*t*) as the two state variables. The input to this system is blood flow *f*(*t*) and the resultant output is the BOLD signal which is the observed signal obtained by the fMRI acquisition equipment. As the blood flow increases, the venous “balloon” is inflated resulting in the expulsion of deoxygenated blood at a greater rate. However, this expulsion is not sufficient enough to counteract the influx of oxygenated blood to the venous compartment resulting in the “early dip” in the BOLD signal. A peak in the signal is observed with a time lag of about 4–6 seconds following the onset of neural activity. Once the distended balloon relaxes, the reduced compartment volume and blood dilution result in another dip following the peak known as the poststimulus undershoot [7].

This plausible model can be modeled by (1). It is represented by a set of nonlinear nonautonomous differential equations. Here *u*(*t*) is a known exogenous neuronal input signal. All the state variables are normalized with respect to their resting values:

(1) s˙εut−1τss−1τff−1,f˙=s,v˙=1τf−v1/α,q˙=1τf1−1−E01/fE0−v1/α−1q.

The neuronal efficacy *ε* represents the increase in flow inducing signal. Signal decay *τ*_*s*_ reflects the decay of neuronal signal spatially, over a few hundred micrometers. Increasing this parameter increases the response of the inflow signal (rCBF) to neuronal input signal and also causes the suppression of undershoot observed in BOLD signal. *τ*_*f*_ denotes the autoregulation time constant. Increasing this parameter also suppresses the undershoot. Transit time *τ*_0_ dictates the dynamics of the BOLD signal and is given by the ratio of resting venous compartment volume and resting flow. In other words, it represents the average time for a blood cell to travel through the balloon like vasculature bundle. Increasing this parameter effectively slows down the signal dynamics resulting in the shifting of the peak of the BOLD signal to the right on the time axis. The stiffness parameter *α* governs the degree of coupled nonlinearity between the flow-volume dynamics of the venous compartment. Finally, the resting oxygen fraction *E*_0_ is an important parameter in understanding the dynamics of the evoked fMRI responses. Increasing this parameter increases the early dip in the BOLD signal. An excellent discussion is presented in [7] by Friston et al. illustrating the hemodynamic model parameters. They computed these parameters using Volterra Kernels up to the second moment and are presented in Table 1.

The process model is constructed from (1) and is given as

(2) $$\begin{array}{c} \dot{x}=f\left(x,\phi ,u,v\right),\;v\sim N\left(0,Q_{v}\right), \end{array}$$

where the state vector $x\left(t\right)={\left[\begin{array}{cccc} f & s & v & q \end{array}\right]}^{T}$ and the model parameter vector is $\phi ={\left[\begin{array}{ccccc} \varepsilon & k_{s} & k_{f} & k_{0} & E_{0} \end{array}\right]}^{T}$. However, since we are performing joint estimation, the state vector and the parameter vector are concatenated together to yield the augmented state vector $x(t)={\left[\begin{array}{ccccccccc} s & f & v & q & \varepsilon & k_{s} & k_{f} & k_{0} & E_{0} \end{array}\right]}^{T}$. *u* and **v** represent the known exogenous input and the process noise, respectively, and are responsible for driving the dynamic process model. *k*_*s*_ = 1/*τ*_*s*_, *k*_*f*_ = 1/*τ*_*f*_, and *k*_0_ = 1/*τ*_0_ have replaced the time constants in (1) for numerical stability. *Q*_*v*_ is the process noise covariance matrix.

The output or the observed BOLD signal is then given by a nonlinear function comprising of venous volume “*v*” and deoxyhemoglobin “*q*.” This is given by

(3) yV0k11−q+k21−qv+k31−v,k1=7E0,k2=2,k3=2E0−0.2,

where *V*_0_ is the resting blood volume fraction. This leads us to the construction of the measurement model:

(4) yhx,w,w~1−eNμ1,σn12+eNμ2,σn22,

where *y* is the observation. The measurement noise *w* can be modeled as a Gaussian mixture (*e*-mixture) where *e* ∈ (0,1) is the mixing parameter, determines the contribution of the non-Gaussian part of the noise, and usually varies from 0.01 to 0.25 [36]. Moreover, the ratio of *σ*_2_/*σ*_1_ is usually in the range of 10 to 10,000 [36, 37]. Due to the inherent tuning flexibility, the *e*-mixture can be used to model several kinds of non-Gaussian distributions.

Equations (2) and (4) can be rewritten as discretized stochastic differential equations (SDEs)

(5) xkfk−1xk−1,uk−1,vk−1, v~N0,Qv,ykhkxk,wk, w~e-mixture.

Equation (5) forms the state-space representation of the BOLD signal. Thus it becomes a data assimilation problem wherein the physiological state variables and model parameters in **x**_*k*_ are estimated from a noisy observation data *y*_*k*_. It should be noted that *V*_0_ and *α* need not be incorporated into the joint filtering framework [35].

### 2.2. Nonlinear Joint Estimation

Both the process and measurement model presented in [30] were linear. We have modified the AGSF algorithm to handle nonlinearity both in the process model and the measurement model. In effect, we have two EKF filters operating in parallel each matched to a Gaussian term of the noise model in (4). We have named the filter as the modified AGSF (MAGSF). The schematic and the algorithm for the filter are presented next.

**Figure 2:**
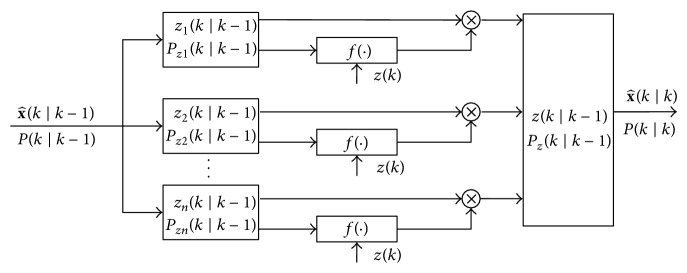
Framework of the MAGSF.

Figure 2 shows the framework of the proposed filter. Unlike the AGSF that has a bank of Kalman filters, the MAGSF has a bank of extended Kalman filters each tuned to a specific term of the Gaussian mixture. The filter is specifically designed to combat Gaussian process noise but non-Gaussian measurement noise. First the filter is initialized with the initial conditions $\hat{x}(0|0)=x(0)$ and *P*(0∣0) = *P*(0) where *P* is the error covariance.

The state estimate computed at the previous time step is propagated through the nonlinear function *f*(·) to yield the state estimate at the current time step. However, note that the data for the current time step is not yet available. The computed state estimate is then used to compute the Jacobian. The computation of Jacobian is a typical step in filters that perform linearization operation. Here we are doing it since the MAGSF employs EKF filters in its core. The covariance is then simply computed by using the Lyapunov or Stein equation [13]. These three steps are represented by the following:

(6) x^k ∣ k−1fx^k−1 ∣ k−1,uk,

(7) Ak−1=dfdxx=x^k ∣ k−1,

(8) Pk ∣ k−1=Ak−1Pk−1 ∣ k−1ATk−1.

Next the state update steps are performed using (9)–(18). First the measurement is predicted for the current time step. This is computed by (9) wherein the individual measurement predictions corresponding to each subfilter are combined together with respective weights to yield the composite measurement prediction. This is then used in (18) for computation of the corrected state estimate for current time step *k*. The weights required in (9) are computed in (12)–(15):

(9) $$\begin{array}{c} \hat{z}\left(k\;|\;k-1\right)=\overset{n}{\underset{i=1}{\sum }}w_{i}\left(k\right)\hat{z_{i}}\left(k\;|\;k-1\right). \end{array}$$

The individual measurement predictions are computed by passing the previously computed states and the mean of the respective Gaussian term constituting the composite non-Gaussian density of the measurement noise, through the nonlinear measurement model equation. This is represented by

(10) $$\begin{array}{c} z_{i}\left(k\;|\;k-1\right)=h\left(\hat{x}\left(k\;|\;k-1\right)\right)+\mu_{i}. \end{array}$$

Next, the measurement error covariance is computed using (11)–(15). Here, too, the measurement error covariance is composed of individual measurement error covariance of each Gaussian term determined by (13). The *R*_*i*_ in (13) is the covariance of the individual Gaussian term:

(11) Pzk ∣ k−1 =∑i=1nPzik ∣ k−1+z~k ∣ k−1z~k ∣ k−1Twik,z~k ∣ k−1=z^k ∣ k−1−z^ik ∣ k−1.

The Jacobian is also computed of the measurement model about the previously computed state estimate as given by (12). Note that |·| denotes matrix determinant and ‖·‖ represents inner product in the *R* ^*m*^ Euclidean space:

(12) $$\begin{array}{c} H\left(k\;|\;k-1\right)={\left.\frac{dh}{dx}\right|}_{x=\hat{x}\left(k\;|\;k-1\right)}, \end{array}$$

(13) Pzik ∣ k−1 =Hk ∣ k−1Pk ∣ k−1Hk ∣ k−1T+Ri,

(14) $$\begin{array}{c} c\left(k\right)=\frac{e^{-{\left\|\tilde{z}\left(k\;|\;k-1\right)\right\|}^{2}/P_{zi}\left(k\;|\;k-1\right)}}{{\left(2\pi \right)}^{m}\left|P_{zi}\left(k\;|\;k-1\right)\right|}\varepsilon_{i}, \end{array}$$

(15) $$\begin{array}{c} w_{i}\left(k\right)=\frac{c\left(k\right)}{\sum_{i=1}^{n}c\left(i\right)}. \end{array}$$

Subsequently, the Kalman gain is computed in (16) making use of the previously computed error covariance, measurement Jacobian, and the measurement error covariance. This is then used to calculate the error covariance for the current time step *k* as given by (17). Finally, the state at time step *k* is computed from (18) making use of the predicted state estimate, Kalman gain, current measurement value, and the measurement estimate:

(16) Kk=Pk ∣ k−1HTk ∣ k−1Pz−1k ∣ k−1,

(17) Pk ∣ k=I−KkHk ∣ k−1Pk ∣ k−1,

(18) x^k ∣ k=x^k ∣ k−1+Kkzk−z^k ∣ k−1.

This sums up the algorithm of the MAGSF. Note that although the MAGSF is employing local filters, that is, EKF, it overall is a global filter. In principle, the more the number of Gaussian terms comprising the composite non-Gaussian density is, the better the estimate will be. Since it is a global filter, the filter will not likely get stuck to a local maxima of a multimodal density.

### 2.3. MAGSF Test-Synthetic Data

**Figure 3:**
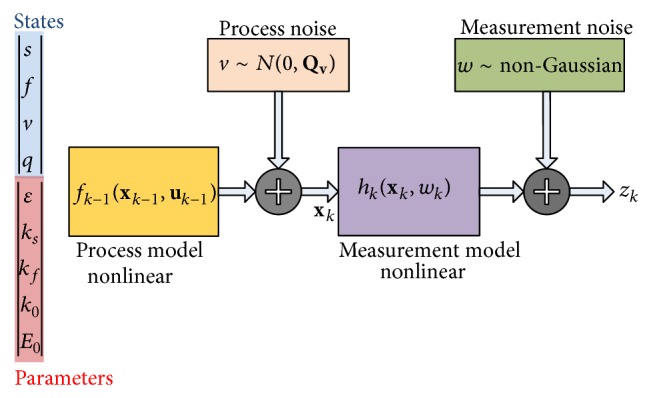
fMRI synthetic data generation scheme.

The data assimilation is first performed employing synthetically produced fMRI data as the ground truth is not available in real data.  Figure 3 shows the scheme used to generate fMRI data. The augmented state vector constitutes the nonlinear process model. Gaussian process noise is added to it before passing the states to the nonlinear measurement model. The measurements are then further corrupted by non-Gaussian noise yielding the synthetic fMRI time series data.

**Figure 4:**
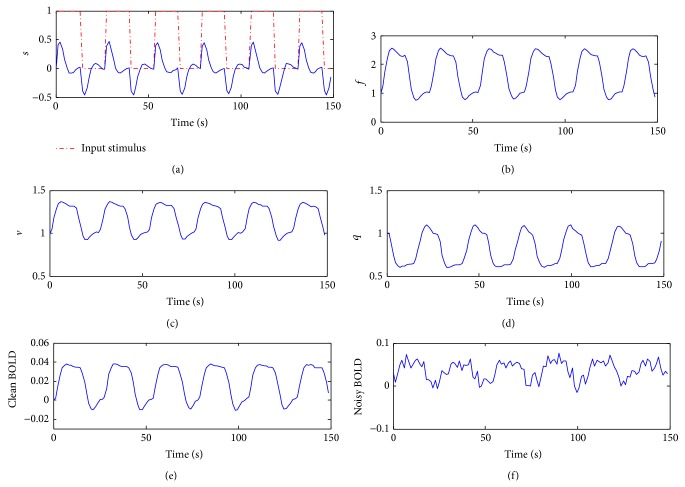
fMRI synthetic data synthesis against a periodic stimulus. *s*, *f*, *v*, and *q* denote flow inducing signal, cerebral blood flow, cerebral blood volume, and deoxyhemoglobin level, respectively.

Figure 4 shows the simulation of hemodynamic model against a periodic stimulus having 13 seconds ON and 13 seconds OFF time and shows the generation of states and the resultant BOLD signal. The model parameters are set as given in Table 1. The process states are corrupted by Gaussian noise and the resultant BOLD signal is corrupted by additive *e*-mixture noise. Each type of noise is proportional to the spectral power density of clean time series data.

The values of *e*, *μ*_1_, *σ*_*n*1_^2^, *μ*_2_, and *σ*_*n*2_^2^ of the *e*-mixture noise were set at 0.01, 0.02, 0.0001, 0.01, and 0.05, respectively. For the process noise, the process noise error covariance matrix is **Q**_**v**_ = 0.0223 · eye(9) where eye(9) represents an identity matrix of size 9, that is, 9 rows and 9 columns. A TR of 1.2 seconds was set and the simulation was run for 150 seconds. The stimulus consisted of a periodic sequence with 13 seconds ON time and 13 seconds OFF time. The ON and OFF values were kept at 1 and 0, respectively. The resultant synthetic data was then applied to both EKF and the MAGSF for joint estimation.

Since we know the ground truth in the case of synthetically produced data, we can determine the error between the true and estimated states. To statistically access the performance of the filters, we have utilized the root-mean-squared-error (RMSE) which is given by

(19) $$\begin{array}{c} \text{RMSE}=\sqrt{\frac{\sum_{i=1}^{k}{\left(\hat{x}-x_{\text{true}}\right)}^{2}}{k}}, \end{array}$$

where *k* is the number of elements in the estimated state vector $\hat{x}$. **x**_true_ contains the ground truth values of the states and parameters.

### 2.4. MAGSF Test-Real Data

Following the data assimilation on synthetic data, estimation was performed on real experimental fMRI data available at [38]. The block design experiment consisted of a human subject performing a simple task of pressing left hand at one time, right hand at the other and resting in between. The data had a temporal resolution of 1.92 seconds. Preprocessing of the data was performed by utilizing SPM8 [39].

**Figure 5:**
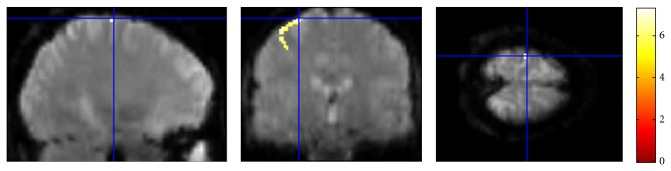
The greatest activated voxels considered for data assimilation.

The origin was aligned to the anterior commissure. Brain activation map was then generated using the respective contrasts and *t*-tests. Threshold level was set at *P* = 0.05. The voxel $\left[\begin{array}{ccc} -28 & -20 & 74 \end{array}\right]$ showing the largest response to stimulation was then selected for analysis.  Figure 5 shows the activation map overlayed onto the brain of the human subject.

Joint estimation was subsequently performed on this time series data. System noise is assumed to be **Q**_**v**_ = 0.071 · eye(9) and measurement noise is modeled as *e*-mixture noise having *e*, *μ*_1_, *σ*_*n*1_^2^, *μ*_2_, and *σ*_*n*2_^2^ set at 0.01, 0, 0.001, −0.01, and 0.05, respectively. The prior distributions of the parameters were set according to values given in Table 1.

## 3. Results and Discussion

**Figure 6:**
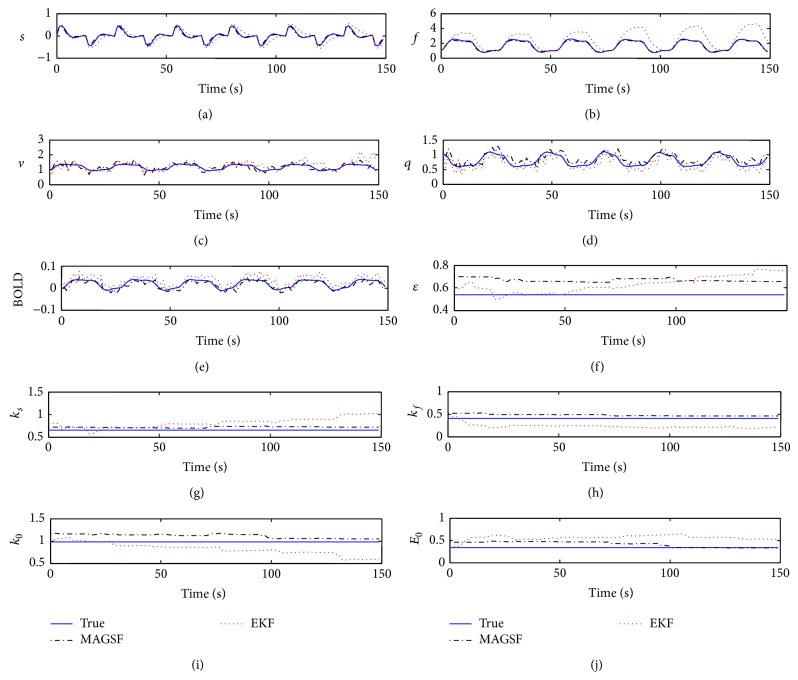
State and parameter estimate results of MAGSF and EKF against synthetic data in impulsive noise. Note that the BOLD signal is not directly estimated from the algorithm. Rather, it is constructed from the estimated states and parameters.

Figure 6 shows the results of the estimates obtained from the data assimilation. Although MAGSF is outperforming EKF in all of the state estimates, the outperformance, however, is significant in Figures 6(a)-6(b). The reconstructed BOLD signal reveals that the EKF is overestimating while the MAGSF is not. This is partly because the noise pdf has a non-zero centre. The EKF assumes that the noise is zero mean whereas the MAGSF does not. Moreover, a closer look at the estimates reveals that the EKF estimates for dHb are biased towards underestimation whereas the MAGSF estimates are not biased.

**Figure 7:**
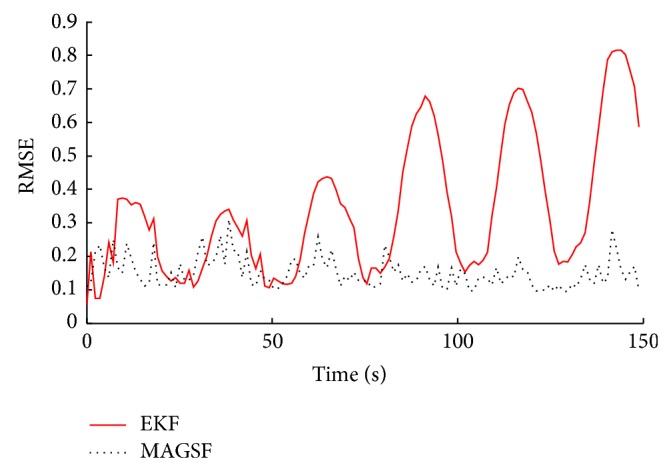
Root-mean-squared-error (RMSE) evolution with time of the joint estimates produced by the two filters applied to synthetic data.

The most important parameter, namely, the resting state oxygen extraction factor, is estimated quite close to the actual value by the MAGSF. In the case of parameter estimates, here, too, MAGSF outperforms the EKF. Most of the parameters estimated by the MAGSF are closer to the ground truth as compared to the ones estimated by the EKF and are less fluctuating.  Figure 7 shows how the RMSE evolves with time as the filters run through the simulation and clearly shows the superior performance of the proposed filter as compared to the EKF. The RMSE of the MAGSF remains around 0.15 throughout the simulation whereas that of EKF keeps on increasing with time or, in other words, the filter seems to diverge.

**Figure 8:**
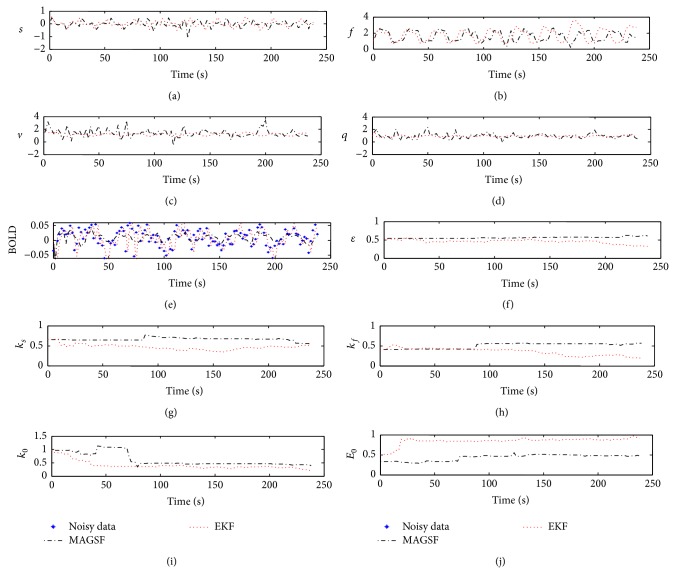
State and parameter estimate results of MAGSF and EKF using real data.

Figure 8 shows the results of the state and parameter estimates for the real data. In this case we do not know the ground truth and can therefore not claim the degree of accuracy of the estimates. We, therefore, present a comparison of the parameter estimates in Table 2 with those reported by previous studies. We see that the parameters estimated by MAGSF are quite close to the rest of the tabulated results. As expected, the results of EKF are quite different. Moreover, the time evolution of the estimates computed by the MASGSF is less fluctuating in nature than the ones produced by EKF as seen in Figure 8.

It is important to correctly estimate the hemodynamic parameters since the parameters serve as the basis for subsequent data analyses such as finding brain activity and making inferences.

Although, presently, it is mostly assumed that the noise affecting the measurements is Gaussian, it will likely change in the future with increase in the understanding of fMRI noise. Our work serves as a motivation for including the assumption of non-Gaussianity in future fMRI based data analyses especially data inversion schemes involving hemodynamic model approaches.

It must be noted that although the MAGSF employs EKF, it itself is a global filter. This prevents it from getting stuck at local minima, hence avoiding false estimates. Moreover, the approach is not limited to *e*-mixture noise. The number of filter banks may be increased until they approximate an arbitrary distribution. It is also worth mentioning that the novel filter proposed is not limited to the application of fMRI but is a general purpose optimal filter and can be applied to other problems such as radar tracking and inertial navigation, and so forth, where the noise may be modeled as non-Gaussian.

## 4. Conclusions

The hemodynamic model provides a plausible explanation for the BOLD signal observed in an fMRI experiment. Deconvolution of noisy fMRI time course yields information essential for understanding the underlying physiological processes occurring inside the brain. Quite often in deconvolution schemes, the process noise and the observation noise are assumed to be Gaussian. Unfortunately, real world systems are plagued by non-Gaussian noise with fMRI being no exception. The celebrated Kalman filter and its variants such as the EKF are designed to work only in Gaussian noise environment. Unsurprisingly a filter designed for Gaussian noise made to work under non-Gaussian environment is likely to perform poorly.

We have proposed a novel nonlinear filter capable of handling non-Gaussianity and named it MAGSF. We then utilized it for performing joint estimation of the hemodynamic model states and parameters under non-Gaussian measurement noise. Due to the absence of ground truth, we first generated synthetic fMRI data and infected it with *e*-mixture measurement noise subsequently utilizing it to compare the performance of the proposed filter against the standard EKF. Analysis was further complemented with the inclusion of real fMRI data. Our results show that the proposed filter outperformed the EKF both in the state and the parameter estimation. This was valid for both the synthetic and the real data. Moreover, our scheme may also be utilized where fMRI data is infected with non-Gaussian noise of an arbitrary shape.

## Figures and Tables

**Table 1:** Statistical distribution of hemodynamic model parameters computed by Friston et al. [7]. *N*(*μ*, *σ* ^2^) represents normal distribution with mean *μ* and variance *σ* ^2^.

Parameter	Statistical distribution
*ε*	*N*(0.54,0.1^2^)
*τ*_*s*_	*N*(1.54,0.25^2^)
*τ*_*f*_	*N*(2.46,0.25^2^)
*τ*_0_	*N*(0.98,0.25^2^)
*α*	*N*(0.33,0.045^2^)
*E*_0_	*N*(0.34,0.1^2^)
*V*_0_	*N*(0.02,0.005^2^)

**Table 2:** Parameter summary and comparison with previous studies.

Parameter	Friston et al. [7]	Hu et al. [17]	Liu and Hu [18]	Our results	
				EKF	MAGSF
*ε*	0.54	0.599	0.55	0.33	0.61
*τ*_*s*_	1.54	1.495	1.55	2.00	1.96
*τ*_*f*_	2.46	2.489	2.45	4.54	1.89
*τ*_0_	0.98	1.087	0.98	4.00	2.08
*E*_0_	0.34	0.527	0.40	0.90	0.49
